# Progress in Medicine

**Published:** 1897-01-16

**Authors:** 


### Progress in Medicine.

### DISEASES OF THE ABDOMINAL VISCERA.

**Peritoneum.**—Byron Robinson[1] gives the results of a careful and extensive examination of the endothelium of the peritoneum. Considerable attention is given to the stomata vera and their surrounding endothelium. He considers that the stomata lead from the general peritoneal cavity into sub-peritoneal lymph spaces. He refers to absorption from the peritoneal cavity, and agrees with other observers, that both organic and finely divided particles of inorganic substances are mainly taken up in the region of the diaphragm. This may have some bearing upon the pathology of the peritoneum. In chronic peritonitis the greatest amount of thickening is almost invariably seen on the under surface of the diaphragm and upper surface of the liver. He quotes Beck as saying that the peritoneal endothelium absorbs material three times as fast as the pleura. Clinical experience accords with such a physiclogical observation. To physicians the danger of septic absorption is known to be far greater in the case of the peritoneum than in the case of the pleura. Barling[2] records three cases of peritoneal hæmorrhage cysts. Two followed injury, and one occurred soon after parturition. One was situated mainly on the right side of the abdomen; the other two were more or less centrally placed in the epigastric region. All recovered after abdominal incision and drainage. The most typical example of the three occurred in a blacksmith, æt. fifty-three. Shortly after receiving a blow in the abdomen he suffered from abdominal discomfort, thought to be indigestion. Some weeks later an epigastric swelling was discovered, which gradually increased in size. On admission to the Birmingham General Hospital a rounded swelling occupied the central area of the abdomen, extending about as far below as it did above the umbilicus. After abdominal incision the swelling was tapped, and 95 oz. of thin brownish-red fluid drawn off. The cyst was then incised, and two or three pints more of similar fluid, with old blood clot, removed. The wound and sinus healed completely, and the patient some months after was in robust health. The fluid removed was examined for pancreatic ferment, but none found. The situation was thought to be probably in the transverse meso-colon. The other cases are, perhaps, less distinctly cases of peritoneal sanguineous cyst. One gave evidence of connection with the kidney, and the other with the pancreas.

**Tuberculous Peritonitis.**—De Minicis[3] had treated four cases of tuberculous peritonitis by the external application of guaiacol. Sometimes a mixture of tincture of iodine and guaiacol was used, sometime a mixture of guaiacol and olive oil. As a result of the applications the temperature fell, and abdominal pain was relieved, and the effusion present diminished, and finally disappeared.

**Intestines.**—Christopher Addison[4] gives a valuable and interesting account of the protozoa found in the fæces. He remarks upon the amœba frequently found in cases of dysentery, and in abscesses of the liver following dysentery, but says that it appears to be still doubtful what part this parasite plays in the production of the disease, since it has been found in the stools of healthy people. Of other parasites, the most important appears to be the Balantidium Coli, which most frequently attacks persons engaged in special trades, chiefly that of the pork butcher. Diarrhœa and passage of blood per rectum may be present which sometimes lead to a fatal ending. The mortality of recorded cases has been 10·77 per cent. Another interesting parasite is the Megastoma entericum, which has occurred in the slimy diarrhœa of children suffering from wasting diseases, sometimes in enormous numbers. The fæces in such cases may contain flocculent lumps consisting of agglutinated masses of parasites. The free parasite occurs in the small intestine, especially in the duodenum, applying itself by a shallow sucker-like depression to the epithelial cells from which it derives its nourishment. It is pear-shaped, and possesses four pairs of flagella. Mongour[5] reports a case in which a large number of intestinal calculi were passed by a young woman, aged thirty-one, who had suffered for six years from digestive disorders. Most of the stones were about the size of orange-seeds, the largest as big as a nut. Their discharge lasted two or three weeks. The concretions were of a yellowish-white colour, and very friable. Some of them presented conical elevations on their surfaces, others were smooth. They were homogeneous on section, and did not contain any central nucleus. Chemical examination showed the stones to be composed principally of carbonate of lime and phosphates of magnesia.

Enteroclysis is again advocated by the *Therapeutic Gazette*[6] in the treatment of summer diarrhœa of children, and a paper by Clemow[7] is reviewed in the same journal, in which the writer states that he has found this method to be of considerable value. The child falls into a quiet sleep after the irrigation; some time elapses before the bowels act again, and, when they do act, the motions are far less offensive than before the operation. Bismuth naptholate is stated by Chaumier[8] to be of value in the treatment of this disease as well as in the diarrhœa of adults; and it is also stated that in two cases of typhoid fever the intestinal disinfection was perfect. Fussell[9] writes upon the value of salol in diarrhœa. He says that four years ago he wrote a paper upon the value of this drug in diarrhœa, and that he wishes to again express his belief in its efficiency. He prescribes salol in a bismuth and chalk mixture.

**False Abdominal Tumours.**—Potain[10] records a case of protrusion in the upper part of the abdomen, simulating a tumour, in a man at forty-three, suffering from heart-disease and ascites. It was in the region of the upper part of the right rectus muscle. The protrusion appeared to be due to local weakening of that muscle. The writer refers to another case seen several years before, where, in a young girl, there was a firm, tender tumour on the epigastrium. An exploratory puncture had been made without drawing off any fluid. Potain thought the tumour possibly a hydated cyst of the liver, but another puncture was made without result. A third puncture made later was also unsuccessful. The patient was then put under chloroform and the tumour disappeared. A false tumour of somewhat different character is referred to by Eersung.[11] In a woman aged thirty-two, an oval elastic tumour the size of a child's head, with a smooth surface, was present, and was thought at first to be a fibroma of the uterus, and afterwards a floating kidney enlarged by growth. Laparotomy showed that the supposed tumour was the sigmoid flexure enormously distended with fæcal matter.

**Pancreas.**—Cayley[12] records a case of acute pancreatitis. A very stout man, æt. thirty, was admitted into the Middlesex Hospital in a state of extreme collapse. His extremities were cold, and his face pinched and slightly cyanosed. The temperature was 98·8 degrees, but fell about an hour later to 95·4. His pulse was 150, scarcely perceptible, and the respirations sighing and irregular. He vomited frequently, and there was severe epigastric pain, associated with great tenderness in this region. He gave a history of having been in good health until three days before admission, when he became constipated, and suffered from pain in the stomach. The pain continued, but not until the day of admission did he become seriously ill. On that day vomiting set in. The advisability of an exploratory abdominal section was considered, but the condition of the patient caused the abandonment of any surgical treatment. Ten hours after admission the patient died. At the autopsy the tissues around the pancreas were found infiltrated with bloody serum, and the pancreas itself enlarged, reddened, and friable. There were spots of fat necrosis in the fat around the pancreas and in the mesentery. A case of acute gangrenous pancreatitis is recorded by Von Bonsdorf.[13] A man, æt. forty-six, was seized with vomiting, abdominal pain, and constipation, which signs were thought to indicate intestinal obstruction. Laparotomy was performed, and a large amount of brownish ascitic fluid escaped. Two days later th patient died, and enlargement of the pancreas was found, with a necrotic mass in its left portion. Fat necrosis of the gland and of the omentum was also present.

[1] Med. Record, July 25, 1896. [2] Birmingham Med. Review, August, 1896. [3] Med. Week, Sept. 18, 1896. [4] Quarterly Med. Journal, July, 1896. [5] American Journ. Med. Sciences, June, 1896; Compte's Rendus de la Société de Biologie., Feb. 28, 1896. [6] Therapeutic Gazette, Aug. 15, 1896. [7] Ibid. [8] Med. Record, Aug. 29, 1896. [9] Therapeutic Gazette, Aug. 15, 1896. [10] Med. Week, July 24, 1896. [11] Ibid., Oct. 16, 1896. [12] B. M. J., July 4, 1896. [13] Am. Med. Surg. Bulletin, Aug. 8, 1896.; Cent. f. Allg. Path., &c., 1896, VII., No. 4.

